# Effect of implementing school meals compared with packed lunches on quality of dietary intake among children aged 7–13 years

**DOI:** 10.1017/jns.2018.29

**Published:** 2019-01-29

**Authors:** Marianne S. Sabinsky, Ulla Toft, Helle M. Sommer, Inge Tetens

**Affiliations:** 1 Division for Diet, Disease Prevention and Toxicology, National Food Institute, Technical University of Denmark, Kgs. Lyngby, Denmark; 2 Research Centre for Prevention and Health, Glostrup University Hospital, Glostrup, Denmark; 3 Data Management and Statistics, SEGES, Danish Agriculture & Food Council, Copenhagen, Denmark; 4 Vitality – Centre for Good Older Lives, Department of Nutrition, Exercise, and Sports, University of Copenhagen, Frederiksberg C, Denmark

**Keywords:** School-based interventions, Dietary interventions, Nutrition programmes, Multilevel analyses, Environmental interventions, Meal IQ, Meal Index of dietary Quality

## Abstract

Strategies are needed to improve the dietary habits of children. The aim of the present study was to evaluate the effect of implementing a school food programme on the dietary quality of lunches consumed by school children aged 7–13 years compared with packed lunches brought from home. A secondary objective was to investigate if a possible effect would differ between the younger children and the older. A quasi-experimental study design with four intervention schools and four matched control schools was conducted. In total, 984 school children participated. Data on packed lunches were collected at baseline. At the 1st follow-up the children in the intervention schools were offered free school meals and at the 2nd follow-up children paid for their school meals. The control group had packed lunches at all measurements. A digital photographic method combined with a Meal Index of dietary Quality (Meal IQ) was used for dietary assessment. Multilevel modelling was employed for data analyses. The quality of dietary intake was improved when free school meals were offered (*P* = 0·004); if the school meals were paid for the use was limited and no difference in change in dietary quality was found (*P* = 0·343). The school food programme had no difference in effect according to age (*P* = 0·083). In conclusion, offering a free school meal had a positive effect on dietary quality of the lunches consumed by school children aged 7–13 years. No effect was measured when the school meals were not provided for free. The dietary effect did not depend on age.

Healthy dietary habits during childhood promote optimal health, growth and cognitive development of the child, and may contribute to the prevention of chronic diseases in later life^(^^1^^,^^2^^)^. Some evidence exists that nutrition behaviours track from childhood into adulthood^(^^3^^,^^4^^)^, and thus it is important to establish healthy dietary habits early in life.

The dietary habits of children in Denmark^(^^5^^)^, as well as for children in other Western countries, call for improvement^(^^6^^)^. Data from the recent Danish National Survey of Dietary Habits and Physical Activity revealed that to meet the official nutrition recommendations^(^^7^^)^ and the food-based dietary guidelines^(^^8^^)^ Danish children should consume less fat, especially saturated fat, less added sugar and increase their intake of fruits, vegetables, whole grains and fish^(^^5^^,^^9^^,^^10^^)^. Thus, there is a need for strategies to promote and provide healthy dietary habits among children. The school has been recognised as an important setting for such a health promotion strategy, because health-related behaviours can be influenced, especially healthy eating habits^(^^11^^–^^14^^)^. The school reaches all school-aged children of diverse ethnic and socio-economic groups and offers an environment that is accessible to all on equal terms.

In Denmark 85 % of children aged 7–14 years eat a packed lunch brought from home during school hours^(^^10^^)^. Studies in Denmark^(^^10^^,^^15^^)^ as well as in other countries^(^^16^^–^^19^^)^ have shown that the dietary quality of packed lunches do not always meet dietary guidelines. Cross-sectional studies have compared the nutritional quality of packed lunches and school meals provided by schools and have reported that children who eat school meals generally have a healthier lunch compared with children who eat a packed lunch^(^^20^^–^^26^^)^. Many studies and reviews on school-based intervention studies have been published. The interventions vary to a great extent in terms of intervention (nutrition education, environmental interventions or multicomponent interventions), duration of the intervention, outcome measures and significance of results^(^^27^^–^^32^^)^. We found only one study substituting the whole lunch meal. Andersen *et al*.^(^^33^^)^ found an increase in the dietary quality of lunches consumed among school children when they had a school meal based on New Nordic Diet principles compared with packed lunches.

In a recent systematic review Brown & Summerbell^(^^29^^)^ found that some interventions appeared to vary in effectiveness according to, for example, the age of the children. Also a Danish cross-sectional study showed that students had different attitudes toward school food programmes. The younger children, representing the 3rd grade (9–10 years), appreciated packed lunches brought from home but children from the 6th (12–13 years) grade were happier with school meals^(^^34^^)^. The prevalence of children who bring their lunch from home decreases with age. The younger school children are comfortable with their packed lunches, but the growing age and youth culture influences the status of the packed lunch among the older school children^(^^35^^)^; thus it is possible that the effect of a school food programme could depend on the age of the children involved.

The aim of the present study was to evaluate the effect of implementing a school food programme on the dietary quality of lunches consumed by school children aged 7–13 years compared with packed lunches brought from home. A secondary objective was to investigate if a possible effect would differ between the younger school children (2nd–3rd grades) and the older (5th–6th grades).

## Methods

### Study sample

We conducted a quasi-experimental study with a pre- and post-intervention design. In 2008, thirty-eight schools received funds from the Danish Food Industry Agency to implement a school food programme. In the first 2 months the school meals were free; this was followed by a period where the school children could buy their school meals. To evaluate the dietary effect of the school food programmes four intervention schools from the thirty-eight schools were selected, taking into account representation of different geographic locations and size of the school. Four schools were selected randomly and afterwards contacted. The four schools all accepted to participate in the study. Four control schools were selected among schools without any school food programme and matched with the four intervention schools with respect to municipality, size (number of children) and families’ social background.

Power calculations using an α level of 0·05 and a β level of 0·8 estimated that fifty children were required in order to detect a difference of 2·29 g saturated fat intake, estimating the intra-class correlation to be 0·02. To examine if a possible nutritional effect of the school food programme was different between students from the 2nd–3rd grades and 5th–6th grades approximately fifty students in each age group in each of the eight participating schools were selected.

**Fig. 1.**
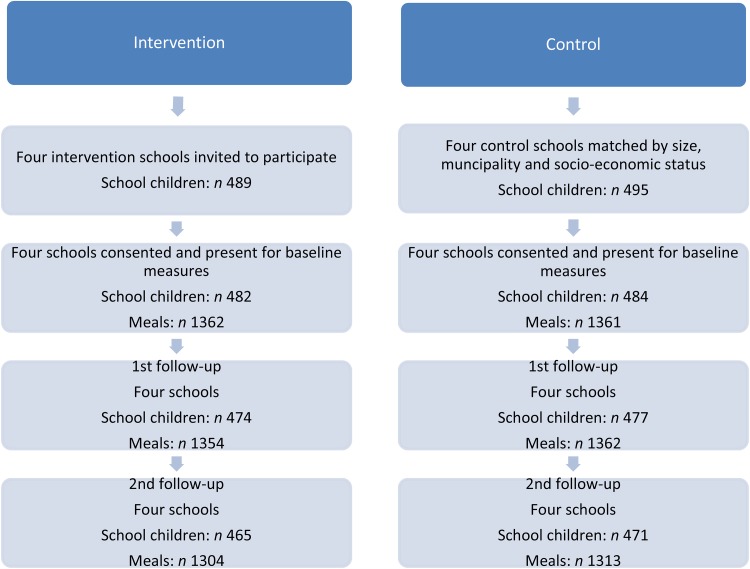
Flow of schools, participants and meals through the study.

Children from forty-six school classes at the eight participating schools were invited to participate in the study. In total 984 children participated, 493 school children from the 2nd and 3rd grades and 491 students from the 5th and 6th grades. For flow of schools, participants and number of meals, see Fig. 1.

### Ethical issues

The present study adheres to Danish ethical standards and has been approved by the Danish Data Protection Agency (reference 2008-54-0497) and reported to the regional ethics committee for the Capital Region of Denmark. They concluded that formal ethics approval was not required because no human biological material was collected.

When schools were invited to participate, written information targeting the school management and the teachers was sent to all schools explaining the implications of participation. Teachers, children and their parents at the participating schools were informed that participation was voluntary, that their information would be used for research purposes only and treated confidentially and of the possibility of withdrawing during any stage of the study. Parents were informed of the study and the possibility of withdrawing their child from the study by written information indicating the purpose of the study, the implication for and involvement of their child. If the parents had further questions, they could call the project manager.

### Intervention – procedure for data collection

**Fig. 2.**
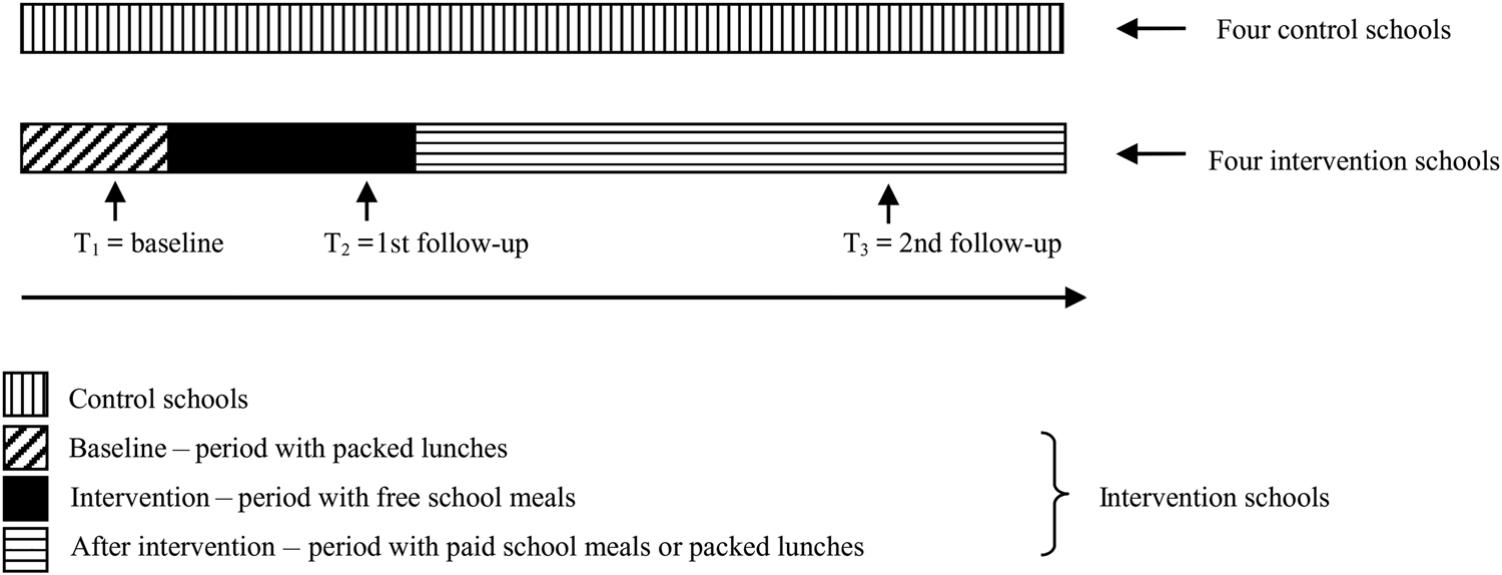
Study design.

At baseline (T1), data on packed lunches were collected in both intervention and control schools. At the 1st follow-up (T2) the control schools still had packed lunches brought from home, and the intervention schools were offered free school meals. At the 2nd follow-up (T3) the controls had packed lunches and at the intervention schools the school meals were no longer for free, so the school children would either have paid school meals or brought packed lunches (Fig. 2). The data were collected successively at the eight schools. At 1 or 2 weeks after data were collected at each of the intervention schools collection of data took place at the matched control school. Baseline data were collected in the weeks before the intervention period began. The 1st follow-up was 8 weeks after baseline and the 2nd follow-up was 6 months after baseline.

Collection of data covered three consecutive days during a week to cover the variability of the lunches over a week.

A validated standardised digital photographic method was used to collect dietary data for the packed lunches or the school meals^(^^36^^)^. At the beginning of the lunch break, the children were asked to place their lunch meals on a plate distributed to them, and all meals were photographed. Where it was difficult to determine what a sandwich contained the child was asked to open the sandwich for viewing. At the end of the lunch break, the plates were again photographed with or without leftovers. In addition, for non-visible food items, the participants were asked questions if the research staff assessed that it would be difficult to see on the digital image. The research staff attended a training session on the use of the digital photographic method before the data were collected.

At the four intervention schools, thirty-one different lunches provided by the schools were served at the 1st and 2nd follow-ups. Recipes and product specifications for lunches provided by the schools were collected. Two of each school meals were bought and the weights of the food items registered. The data on the packed lunches and the school meals were collected during August–December and February–April.

### Assessment of quality of dietary intake

The dietary quality of the lunches was assessed using a validated Meal Index of dietary Quality (Meal IQ), which is a tool that we developed for the purpose. The Meal IQ consists of seven components: total fat, saturated fat, whole grains, snack products, fruit, vegetables and fish, selected with the aim of assessing the overall dietary quality of the lunches. Fruits, vegetables and fish were estimated in g. To estimate total fat, saturated fat, whole grains and snack products in the lunch meals, units were defined in terms of household measures, such as slices, cups and pieces^(^^37^^)^. The total score for the Meal IQ ranged from 0 to 28. Each of the seven components scored from 0 (lack of compliance) to 4 (full compliance), with intermediate scores reflecting levels of attainment towards dietary recommendations.

A database was developed in Microsoft Excel for the dietary assessment of the digital images in order to make the necessary notes on the dietary components in the Meal IQ while watching the digital image. If there were any doubts about the food items on the digital image decisions were made based on consensus between the two digital image analysts; if consensus was not possible the digital image was excluded from the study.

More thorough details about the Meal IQ are given in the previous paper describing the development and validation of the Meal IQ^(^^38^^)^ and the paper on the validation of the digital photographic method^(^^36^^)^.

### Self-reported questionnaires/interviews and anthropometrics

A questionnaire was used to collect data on sociodemographic characteristic of the participating children. The students from the 2nd and 3rd grades were interviewed and the students from the 5th and 6th grades filled out the questionnaires themselves. The majority of the questions used were developed, validated and used in the Pro Children project^(^^39^^)^. The Danish Occupational Social Class (DOSC)^(^^40^^)^ measure was used to assess the social background of the child's family.

At baseline the height and weight of the students were measured to calculate BMI (kg/m^2^). The measures were taken in light clothing and without shoes. Weight was measured to the nearest 0·1 kg using a Soehnle Verona 63749 digital scale; height was registered to the nearest 1·0 cm using a Soehnle 5003 digital height rod.

### Statistical analysis

The dietary effect of the school food programme was examined using the Meal IQ score which were measured as repeated measurements for the same group of children at baseline (T1), 1st follow-up (T2) and 2nd follow-up (T3). The analyses were conducted on the differences of the Meal IQ score compared with baseline by using the following model:
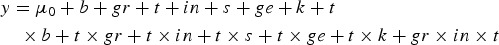
where *y* is the response variable (the difference in Meal IQ score relative to baseline value), *μ*_0_ is the intercept (overall mean), *b* is the BMI value at baseline, *gr* represents the grades (2nd–3rd grades and 5th–6th grades), *t* represents the measurement times (T2 and T3), *in* represents two groups (intervention and control), *s* represents the social status, *ge* represent the sex, *k* is the Meal IQ score at baseline, *t* × *b, t* × *gr, t* × *in, t* × *s, t* × *ge* and *t* × *k* represent the two-way interactions with time, and *t* and *gr* × *in* × *t* represents the three-way interaction term. In addition to the deterministic variables the model given above included a number of stochastic variables which took into account the clustering of children within schools and classes and the repeated measurements of the same child. Thus the following hieratical structure was included in the model:
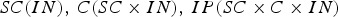
where *SC* represents the schools and is nested with intervention (*IN*), *C* represents the classes and is nested with school and intervention, and *IP* represents a personal index for each child's participation in the study and is nested with school, class and intervention.

The two-way interaction terms were included in the model to test whether the development in the mean changes in Meal IQ score were parallel over time, for example, in intervention and control schools (*t* × *in)*.

Contrasts were constructed from the fitted model to test the particular hypothesis: is a mean change in the quality of dietary intake found when school children eat school meals instead of packed lunches? This was tested at the times of the 1st follow-up and the 2nd follow-up. The estimated mean change values in the contrast were adjusted for other factors of the relevant factors in the model.

Prior to the main analyses baseline tests were conducted to verify that the participating children in the selected schools and classes were not significantly different from each other according to age, sex, BMI, social background of the families and the Meal IQ score.

*P* < 0·05 was considered statistically significant. All reported *P* values were based on two-sided hypotheses. Statistical analyses were carried out using the SAS statistical software package, proc mixed (version 9.2; SAS Institute Inc.).

## Results

### Baseline characteristics of the children

Table 1 shows the characteristics of the participating children at baseline. No significant differences were found between the intervention group and the control group in sociodemographic variables at baseline except for ‘age’ among the younger school children (*P* < 0·0001). This difference occurred due to more 3rd-grade students in the control group. Regarding the quality of the dietary intake (expressed by the Meal IQ score) from the packed lunches brought from home there were no differences between the intervention and the control groups.

**Table 1.** Characteristics and quality of dietary intake (Meal Index of dietary Quality; Meal IQ) of packed lunches in the intervention and control groups at baseline (Mean values and standard deviations; numbers of participants; percentages of participants)

	Intervention		Control		
Characteristics	Mean	sd	Mean	sd	P*
Age (years)	9·65	1·65	9·73	1·59	0·473
*n*	446		438		
Grade					0·748
2nd–3rd	240		246		
5th–6th	242		238		
% 2nd–3rd	49·8		50·8		
*n*	482		484		
Sex					0·248
Girls	234		217		
Boys	248		267		
% Girls	48·5		44·8		
*n*	482		484		
BMI (kg/m^2^)	18·3	2·8	18·4	3·2	0·484
*n*	474		473		
Social class† (*n*)					
I	29		39		0·248
II	14		14		0·991
III	165		179		0·538
IV	177		167		0·620
V	37		29		0·333
VI	40		31		0·295
VII	4		8		0·254
VIII	0		1		0·319
Missing information	16		16		0·991
Meal IQ	11·3	4·8	11·5	5·1	0·241
*n*‡	1362		1361		

* *P* values are based on the *t* test statistic; *P* values for comparison of proportions are based on the *χ*^2^ statistic.

† Danish Occupational Social Class (DOSC) measure^(^^40^^)^.

‡ Number of meals.

### Intervention effect on dietary quality of lunch consumed

Fig. 1 describes the flow of the participating school children and collected meals. Because the response variable is the difference in Meal IQ score relative to baseline value, only the school children participating at baseline were included in the analyses. At T2 the number of children is 951 and at T3 936 children are included in the analyses. In total, data on 8056 lunch meals were included in the analysis, in 2431 cases dietary data were collected from a child during the three measurements collected all three lunch meals, 341 times data were collected on two lunch meals and in eighty-one cases data on one lunch meal were obtained. We excluded 146 meals, because data at baseline were not obtained. Three lunches were excluded from the analyses because consensus between the analysts was not reached about the food items on the digital images.

**Fig. 3.**
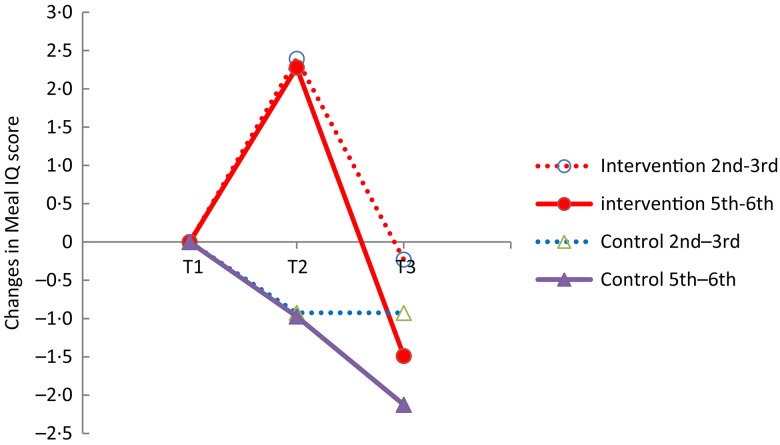
Comparison of changes in Meal Index of dietary Quality (Meal IQ) score between school children in intervention and control schools in 2nd–3rd grades and 5th–6th grades. T1, Baseline; T2, 1st follow up; T3, 2nd follow-up.

Fig. 3 illustrates the development of the changes in the dietary quality of the lunch consumed, expressed by the fitted Meal IQ values, in children in the 2nd–3rd grades and 5th–6th grades in the intervention and control schools at T1, T2 and T3.

A different development over time was seen between the intervention group and the control group illustrated by a significant interaction term *in* × *t* (time × intervention) (*P* < 0·0001) (Table 2). The overall tests of differences in change between children at the intervention schools and the control schools at time points T2 and T3 were investigated. At T2 children in the intervention schools, eating school meals provided by the school, had a significantly improved dietary quality of the lunch consumed relative to children in the control schools, having packed lunches brought from home (*P* = 0·004). At T3, about 4 months after the intervention, no significant difference between the dietary quality of lunch consumed in the intervention and control schools was found (*P* = 0·343). When the children had to buy the school meals the use of the school food programme was limited. At T3 only 7 % of the lunch meals collected were school meals. At two schools the children did not use the opportunity to buy a school meal and at the other two schools, 21 and 6 % of the lunch meals consumed, respectively, were school meals. Overall, only 7 % of the lunch meals collected at the intervention schools at T3 were school meals.

**Table 2.** Significant explanatory variables from the main analysis of effects on changes in dietary quality† (Estimates with their standard errors)

Symbols	Effects	Estimate	se	P*
*μ*_*0*_	Intercept	6·99	0·54	<0·0001
*K*	Meal IQ_baseline_	−0·66	0·03	<0·0001
*In*	+ Intervention	3·13	0·56	0·016
	− Intervention (control group)	0		
*Gr*	Grade (5th–6th)	−0·55	0·30	<0·0001
	Grade (2nd–3rd)	0		
*T*	Time (T3)	−0·93	0·48	<0·0001
*k* × *t*	Time (T2) Meal IQ_baseline_ × time (T3) Meal IQ_baseline_ × time (T2)	0 0·08 0	0·04	0·026
*in* × *t*	Intervention × time (T3) (intervention group)	−2·70	0·28	<0·0001
	Intervention × time (T2) (intervention group)	0		
	Intervention × time (T3) (control group)	0		
*t* × *gr*	Intervention × time (T2) (control group) Time × grade (T3) (5th–6th) Time × grade (T3) (2nd–3rd) Time × grade (T2) (5th–6th) Time × grade (T2) (2nd–3rd)	0 −1·17 0 0 0	0·28	<0·0001

Meal IQ, Meal Index of dietary Quality; T2, 1st follow-up; T3, 2nd follow-up.

* *P* value for type 3 tests for fixed effects.

† The effect estimates of all parameters plus their standard errors are given together with the *P* values (*n* 5333).

The Meal IQ scores of the two age groups divided into 2nd–3rd grades and 5th–6th grades were significantly different at baseline (*P* < 0·0001). The mean Meal IQ scores for the younger and older age group were, respectively, 11·7 (sd 4·5) and 11·1 (sd 5·4). Furthermore, a significant interaction between time and grade was found (*P* < 0·0001) (Table 2), indicating a different development in changes in dietary quality over time depending on age. The three-way interaction term (time × intervention × grade), however, was non-significant (*P* = 0·083), which shows that there is no different effect of the school food programme at T2 and T3 between children in the 2nd–3rd grades and 5th–6th grades. Thus the differences between age groups seen in Fig. 3 are not due to accessibility of school meals.

Table 2 shows the *P* values and the parameter effect estimates of the explanatory variables significantly associated with the change in dietary quality for the final model.

Some of the different effects of age can be explained by the fact that more children in the 5th and 6th grades did not bring a packed lunch or skipped a meal compared with the children in the 2nd and 3rd grades. When this effect *m* (skip a meal: yes/no/sometimes) was accounted for in the model as a new explanatory variable, the variable *gr* (grade) was no longer significant and was taken out of the model since most of the reason for a difference between the grades was explained by this ‘new’ variable.

If a child does not eat a meal at all in the timetabled lunch break the Meal IQ score is 0. These observations of children not eating lunch were included in the multilevel analyses. Analyses were also done where the skipped meals were excluded. These analyses showed the same overall results, that a significant difference between the intervention and control groups was found at time point T2 (*P* = 0·0006) and no difference was detected at T3 (*P* = 0·553).

The positive change in the Meal IQ score at time point T2 (when the school lunches were provided by the schools) is caused by a reduction in total fat and saturated fat and intake of snack products. The consumption of vegetables and fish increased and the intake of whole grains and fruits decreased (Table 3).

**Table 3.** Components of the Meal Index of dietary Quality (Meal IQ) (unadjusted data) at baseline (T1), 1st follow-up (T2) and 2nd follow-up (T3) in the intervention and control groups (Mean values and standard deviations)

	Baseline				1st follow-up				2nd follow-up			
	Intervention (*n* 1362)		Control (*n* 1361)		Intervention (*n* 1382)		Control (*n* 1399)		Intervention (*n* 1343)		Control (*n* 1355)	
	Mean	sd	Mean	sd	Mean	sd	Mean	sd	Mean	sd	Mean	sd
Total fat (units)	−0·19	1·26	−0·15	1·14	0·82	1·03	−0·18	1·14	−0·17	1·17	−0·04	1·03
Saturated fat (units)	1·77	1·38	1·51	1·29	0·80	0·99	1·37	1·26	1·57	1·32	1·20	1·25
Whole grains and potatoes (units)	0·97	0·83	0·89	0·86	0·56	0·87	0·75	0·78	0·80	0·81	0·67	0·77
Snack products (units)	3·39	1·29	3·32	1·37	13·64	0·81	3·22	1·47	3·28	1·44	3·05	1·60
Fish (g)	1·49	5·66	1·46	5·71	4·15	11·9	1·51	6·04	1·17	5·23	1·51	5·94
Fruit (g)	22·1	48·4	27·6	52·5	10·6	31·9	20·9	43·2	15·0	41·5	18·3	42·4
Vegetables (g)	28·9	36·5	29·38	38·0	46·4	41·7	24·4	34·0	24·5	34·0	22·0	33·1
Meal IQ	11·3	4·80	11·55	5·09	13·64	4·02	10·7	5·22	10·4	4·86	10·1	5·56

## Discussion

The study showed that the dietary quality of the lunch eaten at school was improved when Danish school children aged 7–13 years had free school meals instead of packed lunches. When the school meals were not provided for free the use was limited and no difference in dietary effect was found between children at the intervention and control schools. Furthermore, the study showed that there was no different effect of the school food programme according to age group. Most of the reason for the different development in changes in the Meal IQ score between children in the 2nd–3rd grades and 5th–6th grades is explained by more skipped meals in the older age group.

The improved dietary quality when students have school meals is consistent with the results from several cross-sectional studies^(^^19^^–^^26^^,^^41^^–^^55^^)^ including a meta-analysis, on seven studies, where Evans *et al.*^(^^20^^)^ compared British school meals and packed lunches from 1990 to 2007 measuring lunchtime nutrient intake in children aged 5–11 years. The cross-sectional design is weaker than that of the intervention studies according to their ability to provide evidence for causal relationships. To our knowledge our study is the first intervention study to examine if the dietary quality of the lunch consumed was different when school children had school meals instead of packed lunches. Other school-based intervention studies have not exchanged a whole meal, but instead focused on single food groups, for example fruit and vegetables^(^^56^^–^^58^^)^, or nutrients, such as fat^(^^59^^)^ or whole grains^(^^60^^)^.

School-based interventions are heterogeneous in terms of design, participants, intervention, outcomes and duration, making it difficult to generalise about which intervention components are most effective. Van Cauwenberghe *et al*.^(^^27^^)^ conducted a review on forty-two European intervention studies with the purpose of summarising the effectiveness of school-based interventions to promote a healthy diet in children (6–12 years) and adolescents (13–18 years). They concluded that in children (6–12 years) strong evidence was found of effects of multicomponent interventions on fruit and vegetable intake. The overall conclusion was that evidence was found for the effectiveness of especially multicomponent interventions promoting a healthy diet in school-aged children in European Union countries on self-reported dietary behaviour.

De Bourdeaudhuij *et al*.^(^^28^^)^ reviewed the evidence of school-based interventions promoting a healthy diet together with healthy physical activity habits on behavioural determinants, healthy diets and physical activity habits and measures of obesity in primary and secondary school children in Europe. In younger children (6–12 years) the evidence was found to be inconclusive as to multicomponent interventions having a positive impact on child obesity in the European context. Overall they suggest that combining educational and environmental components that focus on both healthy diet and physical activity give better and more relevant effects.

This study attempted to influence eating behaviour in school children via availability (T2) or accessibility (T3) of school meals. Nevertheless, it seems that interventions operating at several levels could be an important strategy when children's dietary habits should be improved. So it is possible that another intervention design, e.g. a multicomponent version, may have improved the sustainability of the dietary effect.

A review^(^^29^^)^ and a cross-sectional study^(^^34^^)^ have reported different results of school food programmes/interventions according to age. However, the differences between age groups in the present study were not due to the school food programme but could be explained by a higher prevalence of skipped meals among children in the 5th and 6th grades compared with children in the 2nd and 3rd grades. Especially at T3 the older children often skipped a lunch meal (10 % did not bring or eat lunch on any of the days at T3); for the younger age group this applied to 0·5 %. The reason why the older school children did not bring a packed lunch could be that they had already eaten their lunch or maybe they eat after school. According to the literature the growing age and youth culture could also explain why packed lunches are not so popular among children in the 5th and 6th grades^(^^35^^)^.

In the present study the quality of the dietary intake from the lunches was no longer significantly different between the intervention and the comparison schools at the 2nd follow-up. This could be explained by the limited use of the school food programme when the school meals were no longer provided for free and thus relatively few school meals (7 %) were represented at the intervention schools at this measurement. This result indicates strongly that the economic perspective of the school food programme is important for the general dietary effects and the sustainability of school food programmes.

In this study the food eaten in the timetabled lunch break was measured. It is not known if the overall dietary quality of the diet for the whole day is influenced by the dietary quality of the lunch or if a poor or healthy dietary intake is compensated for during the rest of the day. Two cross-sectional studies have compared packed lunches and school meals and also measured the whole day's energy and nutrient intake. One study suggested that the differences in intakes were compensated for by other foods consumed during the day, such that daily nutrient intakes were not significantly different^(^^47^^)^ and the other study suggested that the difference according to type of meal persisted assessing the nutrient intake of the whole day^(^^23^^)^. This issue has to be investigated further.

It is a challenge to assess dietary intake among children. Using a validated digital photographic method overcomes the recall problems and difficulties in estimating portion sizes that exist when collecting dietary data on children, and has the positive side effect that it minimises the burden of the respondent^(^^36^^)^. The Meal IQ score that was used, which has been shown to be a valid indicator of the overall dietary quality, was developed with the purpose of being simple, flexible with regard to the different types of meals, and also being sensitive enough to measure relevant differences when children were having school meals instead of packed lunches^(^^38^^)^. The Meal IQ does not give information on the energy or the exact nutrient content of the meals, but as a tool for evaluation of school-based interventions or interventions in other settings it seems very suitable.

The effect of the school food programme resulted in a significant increase in the Meal IQ score of 2·34 points. A challenge when using indices to assess dietary quality is that the total score covers both positive and negative changes. In this study the positive effect of the free school lunches was due to a decrease of total fat, saturated fat and snack products and an increase in the consumption of vegetables and fish. The result also included a decrease in whole grains and fruits. The dietary change shown in this study is of importance. The decrease in saturated fat goes from 1·77 units at baseline (T1) to 0·80 units at the 1st follow-up (T2). A fat unit was defined as 5 g fat. Also, if the fat units are animal-based they were counted and used as an approximation of the content of saturated fat in the meal. More than half of the content of fat in a saturated fat unit comes from saturated fat. A decrease of 0·97 units from the component of saturated fat in the Meal IQ is thus of dietary relevance, and corresponds to a reduction of at least 2·4 g of saturated fat and thus a reduction of 3–4 % of energy from saturated fat among children in this age group. The increase in intake of fish was not very high, going from 1·5 to 4·2 g. Such a change is not of dietary relevance now, but more children have been introduced to fish and that could perhaps have a positive perspective in the future.

A limitation of this study was the randomisation procedure, where a complete randomisation was not possible because the intervention schools were selected among the group of schools receiving funds from the Danish Food Industry Agency for implementing the school food programme. However, the study schools were matched with controls on key variables.

In the present study, multilevel analysis was used, which is a strength, as it takes into account the study design and also the structure of the data. Not all school-based studies have utilised the hierarchical structure of the data (students nested within schools and students within classes) in their statistical analysis, which might have led to biased conclusions regarding the effect of school^(^^61^^,^^62^^)^. We adjusted our analyses for various known or potential confounders, but we cannot exclude confounding through factors that were not considered.

Most Danish school children bring their packed lunch from home and the lunches do not in general meet the dietary guidelines^(^^5^^,^^7^^,^^8^^,^^10^^)^. The results of the present study suggest important national implications for school food programmes as a potential relevant health-promoting strategy which may improve the quality of dietary intake at lunch. However, this requires additional research on how school food programmes can be better implemented, including knowledge about the economic perspective of this area.

In conclusion, the implementation of the school food programme had a positive effect on the dietary quality of the lunches consumed by students aged 7–13 years in the period where the school meals were offered for free, but when the school meals were paid by the parents the use was limited and no overall effect on dietary quality was measured. The dietary effect of the school food programme did not differ between the children in the 2nd–3rd grades and 5th–6th grades.
